# Navigating Ovarian-Adnexal Reporting and Data System Magnetic Resonance Imaging (O-RADS MRI): A Review of Its Evolution, Current Advances, and Persistent Challenges in Ovarian Imaging

**DOI:** 10.7759/cureus.86717

**Published:** 2025-06-25

**Authors:** Rahul Dev, Udit Chauhan, Tripti Prajapati

**Affiliations:** 1 Department of Diagnostic and Interventional Radiology, All India Institute of Medical Sciences, Rishikesh, IND

**Keywords:** adnexal mass lesion, cect, dwi-adc, mri, o-rads mri, time-signal intensity curve, usg

## Abstract

The Ovarian-Adnexal Reporting and Data System magnetic resonance imaging (O-RADS MRI) is a standardized risk stratification system designed to enhance uniform interpretation and reporting of adnexal masses on MRI.

A PubMed search was conducted using the keyword "O-RADS MRI," yielding 61 articles in the search results. After excluding eight articles, 53 articles were selected. Additionally, five articles were identified through a citation search. A total of 58 articles were included in this literature review.

Ultrasonography (USG) is the primary imaging modality used for evaluating adnexal lesions, with MRI reserved for cases that require further evaluation. Based on both USG and MRI imaging, various scores are assigned to a particular lesion. Contrast imaging, utilizing both ultrasound USG and MRI, is employed for the better characterization of lesions in terms of internal morphology, with a primary focus on solid components. Additionally, internal septation, wall characteristics, and other soft tissue components, including fat, fibrous tissue, and blood products, are also evaluated. Advanced MRI techniques, such as diffusion-weighted imaging and dynamic contrast-enhanced sequences, also help refine the final O-RADS score of a lesion. Contrast-Enhanced Computed Tomography (CECT) plays a predominant role in evaluating metastatic disease and has been established in five cases.

This review article provides a comprehensive overview of O-RADS MRI, addressing its development, key imaging features, and practical application in a clinical setup. We discuss the diagnostic performance of O-RADS MRI in differentiating benign from malignant adnexal lesions, exploring its strengths in reducing inter-observer variability and guiding patient management. We also highlighted various comparative studies and trials that have shaped the evolution of the O-RADS MRI system over time. Furthermore, this article highlights the challenges associated with implementing O-RADS MRI, including potential pitfalls in interpretation, corroborations, and discordance with other imaging modalities, particularly USG, as well as the need for further validation studies.

## Introduction and background

Ovaries usually bear multiple follicles during the premenopausal age. A dominant follicle is formed during each menstrual cycle, denoted as the corpus luteum, after ovulation. Simple ovarian cysts up to 3 cm in dimension are usually considered a form of the dominant follicle [1].

During the postmenopausal stage, the ovaries shrink, and the number of follicles in the ovaries reduces progressively. Benign cysts can occasionally be seen in postmenopausal women and are commonly denoted as an adnexal mass lesion (AL) since a minority of these arise from extra-ovarian structures.

ALs are the most frequently occurring non-physiological gynecological abnormalities that require imaging at an increasing frequency. Imaging these lesions is required first to ascertain benignity and differentiate them from malignant counterparts. AL of ovarian origin can be classified into epithelial, germ cell, and sex cord-stromal histological subtypes [2]. Epithelial tumors are the most common and can be subdivided into serous and mucinous histological variants, as well as types 1 and 2, based on genetic derivation [3]. Type 1 epithelial tumors are low grade and tend to have a good prognosis, largely due to their slow growth and confinement to ovarian tissue despite their large dimensions. Type 2 tumors are high-grade variants that show rapid growth and late presentation, resulting in a poor prognosis. Unfortunately, type 2 tumors contribute around two-thirds of all epithelial ovarian tumors [3].

Most ALs are benign, with a minority being malignant; the demarcation between the two is relatively straightforward in the majority. In a minority of cases, this demarcation becomes an uphill task [1]. It requires imaging across multiple modalities and backgrounds in both clinical and laboratory contexts, as histopathology is not a valid option in every case. Percutaneous ultrasonography (USG) or computed tomography (CT)-guided biopsy is not recommended as the tissue yield is poor, a sinister lesion is missing, and there is a high risk of peritoneal seeding. Biopsy risks up or downstaging a localized lesion simultaneously [4,5].

The overall mortality of malignant ovarian lesions is around 5%, with a 50% five-year survival rate [6]. Risk stratification of these lesions and follow-up for serial progression or assessing response to treatment is also required if they are malignant [7].

## Review

Methodology

A PubMed search was done using the keyword "O-RADS MRI." A total of 61 articles were shown in the search results. Eight articles were excluded, of which three were due to the non-availability of full text, three were errata of other articles, and two were in languages other than English (one in Korean and one in French). After these exclusions, the remaining 53 articles were included. Five articles were also included after citation searches. A total of 58 articles were included in writing this literature review.

PRISMA Flow Diagram

**Figure 1:**
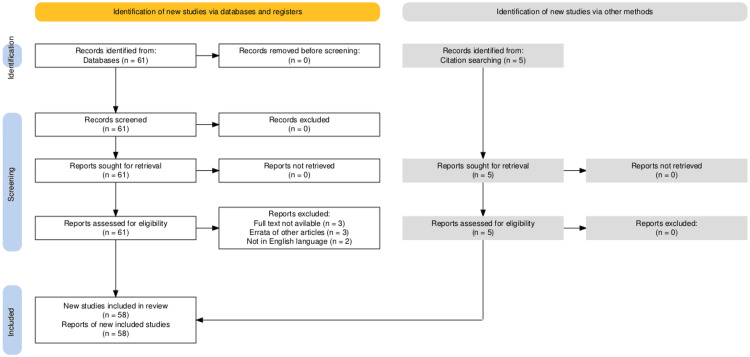
PRISMA flowchart summarizes the study selection process. PRISMA, Preferred Reporting Items for Systematic Reviews and Meta-Analyses

The selection process for studies included in the review is outlined in the Preferred Reporting Items for Systematic Reviews and Meta-Analyses (PRISMA) flow diagram, as shown in Figure 1.

Imaging modalities

Ultrasonography

USG is the first imaging modality used to assess AL [1,8]. It is widely available, relatively easy to operate and interpret, non-invasive, flexible for multiple scans, and devoid of risks associated with radiation and contrast media administration. Despite these advantages, a significant proportion of AL, ranging from 10% to 30%, remains indeterminate after scanning on one or multiple occasions [9]. Atypical imaging appearances and less experienced operators are common contributors. Several USG-based scoring systems have been developed, but their practical reproducibility remains to be observed [4]. The risk of malignancy assessed by transvaginal ultrasonography (TVUS) was also not certain in one study [10]. However, the 2022 Ovarian-Adnexal Reporting and Data System (O-RADS) update recommended TVUS for the evaluation of AL, with the added caveat that the adequacy of transabdominal ultrasonography (TAS) alone should be considered in cases where TVUS is not feasible or inadequate [11,12]. The transrectal approach may be beneficial when both transabdominal sonography (TAS) and transvaginal sonography (TVS) are suboptimal.

The optimization of grayscale and color Doppler parameters is crucial when evaluating AL. Gain, depth, and width are of prime importance in grayscale parameters. In contrast, for the optimization of color gain, the size of the color box, velocity, and frequency range are important [12].

O-RADS USG comprises six risk assessment categories similar to those in O-RADS magnetic resonance imaging (MRI). According to O-RADS, a USG score of 1 indicates normal ovaries, a score of 2 carries a malignancy risk of less than 1%, a score of 3 ranges from 1% to 10%, a score of 4 ranges from 10% to 50%, and a score of 5 indicates a risk of more than 50% [13]. The negative predictive value (NPV) of a benign-appearing AL is as high as 99% [14].

One study compared O-RADS USG with O-RADS MRI [10]. USG and MRI combined showed the greatest diagnostic accuracy for O-RADS score 4 and higher lesions in identifying malignant lesions. If only ALs with O-RADS USG scores of 4 and 5 are referred for surgical management, it could also potentially reduce the number of unwanted surgeries [1,14]. MRI is more specific than USG for lesions up to 5 cm, as it upgrades and downgrades lesions interpreted on USG. The percentage of malignant cases was higher in postmenopausal women than in premenopausal women, partly due to the inclusion of larger lesions beyond 5 cm in size [1,2].

The study by Wang et al. also compared diagnostic validity among O-RADS USG, O-RADS USG with contrast-enhanced ultrasonography (CEUSG) and Assessment of Different Neoplasia in the Adnexa (ADNEX) systems. The highest sensitivity and NPV were found for O-RADS USG. In contrast, the highest specificity and positive predictive value (PPV) were observed for the ADNEX score, which also demonstrated the highest accuracy [1]. O-RADS USG, when combined with CEUSG, increased accuracy, specificity, and PPV.

The major discrepancy between USG and MRI scores was seen for lesions with the cutoff size of 10 cm as O-RADS score 2 lesions are upgraded to O-RADS score 3 once they attain a size of more than 10 cm on USG, while they retain O-RADS MRI score of 2, leading to a downgrading of the score on MRI [14,15,16]. Conversely, lesions greater than 10 cm in size may also be downgraded on USG if the solid component is eccentrically located. The 2019 update of the Society of Radiologists in Ultrasound (SRU) used the longest dimension of the lesion as the threshold [16]. O-RADS USG amendment recommends measuring the sum of all three dimensions to calculate the true dimension of the lesion, along with selecting high-risk features to increase sensitivity [16]. The rationale for this sum measurement was the differences in measurement technique among operators and compression from the probe, which potentially deformed the lesion [12]. CEUSG has also evaluated AL on walls, septations, and solid components [1].

Contrast-Enhanced Computed Tomography (CECT)

CECT of the chest, abdomen, and pelvis is also integral to the metastatic workup, with the added advantages of a low-cost burden and widespread availability [14,17,18]. Positive oral contrast helps increase the conspicuity of serosal and mesenteric deposits, a concern in cases of debulking surgery. Deposits in the mesenteric root, long segment serosal or bowel involvement, including but not limited to the stomach and duodenum, head or body of pancreas, involvement of the celiac trunk, hepatic or left gastric arteries, preclude surgical management due to associated morbidity [19]. Deposits in perifissural locations, on the liver surface, and at the splenic hilum require specialized surgical expertise [17]. Lesions, such as simple cysts and dermoid, can be confidently diagnosed on CECT by their characteristic attenuation values; however, demarcation between hemorrhagic contents and solid components can be difficult. In addition, a large hemorrhagic component may also obscure the coexisting solid component, highlighting the importance of MRI [14].

Magnetic Resonance Imaging (MRI)

MRI has emerged as the gold standard modality for evaluating AL, significantly improving diagnostic accuracy and lesion characterization with gadolinium-enhanced imaging. The large field of view (FOV) and excellent soft tissue contrast enable more accurate characterization AL, complemented by multiplanar capability, which aids in the evaluation of other abdominal organs for metastatic workup [1,7,20,21,22]. The addition of diffusion-weighted imaging apparent diffusion coefficient (DWI-ADC) and dynamic contrast-enhanced MRI (DCE-MRI) further enhances the precision of the diagnosis, instilling confidence in its use. DCE-MRI enhancement curves were also seen to correlate with the expression of vascular biomarkers by the tumoral cells [20]. DCE-MRI can be both technically challenging and time-consuming, which undermines its practical applicability [7].

MRI is recommended to evaluate all ultrasound-indeterminate adnexal lesions in up to 30% of cases with strong evidence, especially for lesions with an O-RADS ultrasound score of 3 and 4, most commonly seen as cystadenomas and cystadenofibromas (CAFs), hemorrhagic or proteinaceous cysts, and unilocular cysts in postmenopausal women [21,23]. This capability of MRI is primarily due to its ability to accurately identify septations and fibrous tissue, with the septations being considered a high-risk feature, a limitation for USG [8,17,24,25,26]. In another study, MRI was found to accurately diagnose sonographically indeterminate lesions in up to 93% of cases [27]. A unicentric ADNEX study, retrospectively evaluated 394 USG indeterminate AL, led to the introduction of a scoring system in 2013 [7]. This study was further validated over five years in a prospective, multicenter MRI study, EURAD [20,28]. Paradoxically, the evidence generated by pathological diagnosis is moderate [17]. MRI is also preferred over CECT in patients with iodine allergy, renal dysfunction, and younger or pregnant patients [17].

MRI also correctly identified two cases of normal ovaries misdiagnosed as AL [25]. MRI also provides flexibility in discussing with surgical colleagues in multidisciplinary meetings, leading to better collaboration and decision-making [13,25].

The patient should fast for three to four hours, administer an antiperistaltic agent, and empty the urinary bladder 30 minutes before the procedure [21,27,29,30]. A pelvic phased array coil centered on the pelvis is a requisite. A body coil with a phased array can also be used with supine patient positioning and should be firmly strapped across the pelvis. A body coil has the added advantage of allowing for the identification of other organs, which can lead to the detection of hydronephrosis, abdominal lymphadenopathy, or peritoneal deposits [29]. If the patient cannot lie supine, they can be positioned in a lateral decubitus position.

The MRI diagnostic algorithm consists of various steps, namely confirming the ovarian origin of AL; the morphology, including its size, shape, and contour; signal intensity (SI) on T1 and T2 with and without fat suppression sequences; DWI-ADC appearance; assessment for any enhancing septae within; and evaluation of the solid component on postcontrast images, either as a single sequence or by including DCE-MRI, and any extra-ovarian findings if present [13,17,26,29].

T1 and T2, DWI-ADC, and pre-and post-contrast T1 fat-saturated sequences with subtraction are essential for detecting macroscopic fat. In and opposed phase, T1 sequences detect microscopic fat [21]. DWI should use b-values of 0 or 50 and 800-1200 s/mm^2^ to obviate the T2 shine-through effect [26,30]. Post-contrast imaging can be acquired preferably as dynamic, with the acquisition of a time-SI curve (TSIC), or as a single acquisition following contrast administration [13]. TSIC images are typically acquired every 10-15 seconds, starting 10 seconds post-injection, with a total acquisition time of 180-240 seconds. This results in a temporal resolution of 15 seconds or less and a spatial resolution of 3 mm [14,21,26,30,31].

The conventional sequences are to be acquired in two planes, namely, axial and coronal or sagittal, with a slice thickness of 3-4 mm and an optional overlap of 1 mm [27]. The area acquired in the axial sequence is from the level of the kidney to the symphysis pubis. For the sagittal sequence, the area acquired is from one hip to the other [32]. If abdomen imaging is also required, the upper limit starts from the diaphragm. Tissue characterization is performed on all sequences across various planes, including the T2 axial upper abdomen sequence, to scrutinize abdominal structures and use DWI-ADC to identify pathological lymph nodes [33].

Magnetic Resonance Spectroscopy (MRS)

The choline peak is typically found in most malignant AL when MRS is performed correctly with high sensitivity and 100% specificity [34]. In all cases of elevated choline peak, there was a concomitant elevation of lipid peak. Benign and borderline AL showed lipid peaks but lacked choline peaks.

18F Fluorodeoxyglucose Positron emission tomography (18F FDG-PET)

AL shows variable amounts of glucose metabolism depending on malignant potential, with benign lesions exhibiting lower metabolism, malignant lesions showing higher metabolism, and borderline lesions displaying intermediate metabolism levels. Glucose metabolism was measured in terms of peak standardized uptake values (SUVp) [18]. With a cutoff SUVp of 1.76, 75% of lesions were malignant. False-positive cases were found to have borderline serous, mucinous, or endometrioid histology. False-negative cases were attributed to clear cells, granulosa cells, and mucinous carcinoma.

SUVp was also corroborated by O-RADS MRI and CECT findings. SUVp values were not considered in cases with an O-RADS MRI score of 3 or less. For the O-RADS MRI score of 4, an SUVp value of 1.76 was used as a cutoff to differentiate between benign and malignant lesions, with lower values indicating benign lesions and higher values indicating malignant lesions [18]. Similarly, if CECT labeled an AL as benign, then SUVp values did not contribute to defining the nature of the AL. On the contrary, if the AL is labeled as malignant on CECT, the SUVp cutoff value of 1.76 discriminated between benign and malignant lesions.

AL represents the most frequently occurring diagnostic dilemma in gynecological imaging. This dilemma is further complicated by the presence of AL across all age groups, with a large spectrum ranging from benign to malignant, including intervening lesions of variable characteristics. Traditionally, benign AL is smaller, typically seen in younger age groups, whereas larger size and older age groups are more commonly associated with malignant counterparts. The most commonly encountered benign ALs are hemorrhagic cysts, endometriomas, and dermoid cysts. Malignant AL includes serous and endometroid carcinoma and metastasis [35].

Tissue characteristics

Solid Tissue

The presence of solid tissue, which enhances, is the most crucial feature within an AL and potentially indicates the malignant nature of the lesion. The size of the solid tissue in both long and short dimensions showed a significant correlation with malignant nature [23]. Solid tissue is any solid component displaying objective contrast enhancement and morphological features like irregular walls, internal septations, mural nodules, papillary projections, or frank discernible solid tissue [13,27,36]. On the contrary, the definition of a large solid component does not include the above morphological features. A solid lesion comprises 80% or more of the total lesion, and a purely solid lesion comprises 100% [30,31,37]. Normal ovarian stromal tissue, mucin, blood clots, fat, cartilage, bone, and hair do not represent solid tissue [11,23]. When the solid component is small, its morphology becomes significant, as evidenced by papillary projections in both borderline and invasive types [23].

In contrast, mural nodules are exclusive to invasive AL [13,32]. From a histological perspective, serous or mucinous borderline lesions can be differentiated by the size of the papillary projection, with those less than 3 mm being found more frequently in mucinous types and those greater than 3 mm being seen more often in serous types. Exophytic papillary projections are exclusive to serous histology [16,32]. When the solid tissue is very small, a thin T2 post-contrast sequence can help identify it [32]. Thin-enhancing walls, septations, papillary projections, and non-enhancing debris are not considered solid tissue [3,30]. Debris can be differentiated from solid tissue based on the lack of color flow and the movement of debris in response to transducer pressure; equivocal cases are to be labeled as solid or evaluated with CEUSG [1,12].

Mural Nodule

The mural nodule is defined as a solid nodular component greater than 3 mm in size, with an obtuse angle to the cyst wall and convex outer margins [37]. It can arise from either the wall or septa of the AL [36]. In contrast, papillary projections bear a fibrous stem and a central vessel, forming an acute angle with the cyst wall [32]. In fat-containing lesions, a Rokitansky nodule (RN) is a solid, luminal projecting component with smooth contours and a convex interface with the lesional wall, although it is not identified as solid tissue [33,38].

Septal Morphology

Septae may be single, few (less than three), or multiple (more than three), thin or thick, smooth or irregular in appearance. Irregular septae show areas of focal thickening and correspond to small papillary projections [32,37]. The term "pseudo nodular thickening" was introduced for type II CAF by Avesani et al. [36].

Intralesional Fat

Fat within a lesion may be macroscopic or microscopic. On USG, fat appears as a hyperechoic component, with shadowing occupying part of the entire lesion [11]. Macroscopic fat appears hyperintense on T1 and T2 sequences and hypointense on fat-suppressed sequences. Microscopic fat can be detected only on chemical shift sequences, not by visual examination [13].

O-RADS MRI designated score of a fat-containing lesion is 2 for those devoid of solid components and 4 for those containing solid components [31,33]. The fat component within an AL may have a variable distribution spectrum ranging from pure fat and fat-fluid levels to focal or diffuse fat areas and fat balls [38]. The most common fat-containing lesions are mature, followed by immature cystic teratomas [3]. Cheng et al. observed in their study that certain features help differentiate benign from malignant fat-containing lesions. Larger lesions in patients younger than 18 years of age, with a large soft tissue component of at least 25%, scattered intralesional fat distribution, irregular margins, solid central enhancement, and a solid component projecting beyond the lesion wall, have a higher propensity for a malignant nature [39]. Benign lesions tend to be smaller with small soft tissue components, showing pure or focal fat, fat-fluid levels, and fat balls with peripheral enhancement [38]. Misinterpretation of the solid component in a teratoma led to upstaging 14 ORADS MRI score 2 lesions to score 4 [40].

T1 and T2 SI

Depending on the T2 SI of the solid component, any lesion may be categorized as low, intermediate, or high, with the low and intermediate SI comparison being the iliopsoas muscle and cerebrospinal fluid (CSF) to designate a lesion as hyperintense. The corresponding reference for the T1 sequence is the iliopsoas muscle and fat [30,31,36]. The cystic component is assessed on T2 and fat-suppressed T1 sequences and classified as having fluid, fat, infected, or hemorrhagic contents, and whether the above contents are uniform or non-uniform within a cyst or locule of the cyst. T1 hyperintensity was defined as a lesion signal higher than that of the pubic symphysis fatty marrow or fat elsewhere. T2 hyperintensity was defined as a signal higher than that of the outer myometrium in at least half of the lesion or higher than CSF [34].

DWI-ADC

DWI has been proven to increase the diagnostic performance of O-RADS MRI scores by up to 15% in one study [4,32]. DWI signal is assessed with the highest b-value (1000 s/mm^2^) by comparing the signal of the solid component in the lesion with the fluid contents under view. The low signal is defined as being comparable to urine or CSF, intermediate when the signal falls between fluid and uterine endometrium, and high when it exceeds the endometrium [30,37]. ADC values correlate with the cellularity of a lesion and are inversely related to progressively reducing ADC values as one progresses from benign to borderline to malignant AL [8]. ADC values of solid components were lower in benign lesions and higher in borderline and invasive lesions [41]. Solid component ADC cutoff value of 1.411 x 10^-3^ mm^2^/s was used to restrict O-RADS MRI score 3 from 4 lesions, and a cutoff value of either 1.08 x 10^-3^ mm^2^/s or 0.849 x 10^-3^ mm^2^/s was used to restrict O-RADS MRI score 4 from five lesions [8,37]. Malignant lesions showed higher DWI SI; however, ADC values did not show a significant difference between benign and malignant lesions [23]. The authors explained this contradictory observation as being due to histological heterogeneity, the inclusion of borderline tumors, the patient cohort, and technical factors [23].

On the contrary, ADC values of intralesional septae or papillary could not be assessed due to the smaller size of the solid components in these lesions [37]. Although corresponding ADC map values are obtained, which provide greater confidence in assessing diffusion restriction, these are not incorporated in O-RADS MRI [36].

A study by Elshetry et al. established the diagnostic performance of mean ADC measurements of solid-enhancing components when read in conjunction with O-RADS MRI. The study found an optimal ADC cutoff value of less than or equal to 1.08 × 10^-3^ mm^2^/s with an O-RADS MRI score of greater than 3 for labeling a lesion as malignant. The corresponding parameters for benign lesions were ADC cutoff values of more than 1.08 × 10-3 mm2/s with an O-RADS MRI score of 2 or 3. Lesions not confirming the above parameters were designated as indeterminate [41].

Similar values were reported by Lin et al., with a cutoff value of 1.27 x 10^-3^ mm^2^/s to differentiate benign from malignant lesions. The mean ADC values of benign and malignant lesions were 2.09 × 10^-3^ mm^2^/s and 0.94 × 10^-3^ mm^2^/s, respectively [34].

When combined, mean ADC and O-RADS MRI measurements were compared with O-RADS MRI or mean ADC alone; the combined assessment provided the advantage of the increase in true negatives and a decrease in false positive cases. The false-positive cases of AL were attributed to cystic components within solid lesions, resulting in higher mean ADC values [42,43]. In contrast, a few benign ALs, such as fibroma, teratoma, and endometrioma, showed low mean ADC values [41]. The majority of reassigned cases had an O-RADS MRI score of 4.

Amide proton transfer-weighted MRI (APTW-MRI) is a novel technique intended for evaluating the cystic component of an AL. It is based on the transfer of saturation from amide protons in the cystic biochemical milieu of an AL to water protons by noncovalent interaction at the molecular level [44]. Higher APTW-MRI values were observed in malignant lesions, likely due to the higher viscosity of the fluid components, which indirectly indicates a higher protein or amino acid concentration. Similarly, hemorrhagic lesions, such as endometrioma, showed higher APTW-MRI values in the early stages than in the late stages, probably due to the lysis of globin protein.

Interestingly, ADC values of the less significant cystic component were evaluated in one study to subdivide O-RADS MRI score 4 lesions, as a mean value of more than 1.69 × 10^-3^ mm^2^/s was associated with a fivefold risk of the lesion being malignant [23,45].

The addition of DWI-ADC reassigned categories of AL, with many lesions being upgraded from score 4 to 5 and fewer downgraded from score 4 to 3 and vice versa, which agreed with histopathological results [8]. Also, in this same study, assigning two ADC values could further subcategorize risk stratification into low and intermediate risk for scores 3 and 4. Rizzo et al. also emphasized the value of DWI-ADC, which can be a discriminator separating score 4 lesions into 4a and 4b [46].

Enhancement

Enhancement within a lesion can be assessed by visual comparison between pre- and post-contrast images or, more effectively, by applying subtraction imaging, which offers the advantage of detecting smaller and more subtle enhancing areas within a lesion. Enhancement within a solid tissue has a high PPV of up to 90%. Conversely, lack of enhancement conveys an NPV of up to 98%. Subtraction imaging is the only assessment technique for enhancement within T1 hyperintense lesions [13,14,21].

Time-SI Curve (TSIC)

TSIC is generated using the perfusion technique for lesions bearing a distinct solid component, the size of which allows placement of a circular region of interest (ROI) for accurate assessment. The acquisition of precontrast sequences is necessary to avoid misinterpretation of TSIC [47]. Besides T2-DWI dark lesions and cases of peritoneal or omental metastasis (O-RADS MRI score 2 and 5 lesions), all lesions require evaluation with TSIC [48].

The visual assessment (VA) of enhancement was compared with TSIC curves, and TSIC analysis proved to be much more accurate than VA in evaluating the enhancement of an AL. TSIC showed higher sensitivity, specificity, and overall accuracy than VA in identifying malignant lesions [48].

The VA was categorized into low, intermediate, and high categories based on the degree of visual enhancement, which was less than, equal to, or greater than that of the myometrium at 30-40 seconds post-contrast injection [13,31]. TSIC was divided into low, intermediate, and high-risk categories for evaluation enhancement at 30 and 60 seconds [29,33,48].

On the contrary, Wu et al. compared non-DCE-MRI for stratification of AL with evaluation of enhancement at 30, 60, and 150 seconds to differentiate between scores 4 and 5 lesions [7].

In TSIC, three curves are drawn depending on the enhancement pattern, with major demarcating features being the initial slope of enhancement and the presence of the shoulder [30]. TSIC type 1 shows a negligible or very gentle slope, with TSIC type 2 exhibiting a gentle or comparable slope to myometrial reference enhancement, preceded by a shoulder and a plateau [29]. TSIC type 3 shows a slope steeper than the myometrial reference, indicating earlier and higher enhancement compared to the myometrial reference [29,30,33,36,48,49].

TSIC type 2 corresponded to low and intermediate-risk VA categories, and TSIC type 3 corresponded to high-risk VA categories. TSIC is valuable in cases demonstrating mild diffusion restriction or those with no appreciable T2 hypointensity in the solid components [36]. Normal ovaries can simultaneously show TSIC type or even 3 curves and diffusion restriction [34,47]. TSIC cannot be drawn for lesions devoid of a solid component, lesions hypointense on both T2 and DWI sequences, and patients whose factors preclude gadolinium administration. One ROI is placed on the lesion's earliest discernible enhancing solid component, with a second ROI on the outer myometrium as a reference point [8,30,47,48]. Uterine lesions, such as leiomyoma, should be avoided for the placement of ROI.

TSIC type 1 signified benign lesions in the majority. However, exceptions do occur, with 1 in 77 O-RADS MRI scoring 3 lesions, indicating that the TSIC type 1 curve was a malignant, clear cell carcinoma borderline lesion in two studies and cystadenocarcinoma and metastasis in another [31,35,48]. Borderline lesions were also assigned TSIC type 1 in the same study. TSIC type 2 raises an intermediate risk for AL, seen in both benign and malignant lesions [8]. TSIC type 3 is strongly associated with malignant lesions; however, teratoma, CAF, and struma ovarii have also been shown to exhibit this high-risk curve, citing false-positive assertions [35,48].

TSIC identified malignant lesions in up to 96%, borderline lesions in up to 87%, and benign lesions in up to 68% of cases. The corresponding values for VA were 76%, 50%, and 81%. TSIC added up to 26% more correct diagnoses over VA, the vast majority being benign lesions [48]. VA alone cannot assign an AL a score of 3, which requires further corroboration with TSIC [30].

Scoring system

O-RADS USG

O-RADS USG, published in 2020, was designed to enhance the characterization of an AL while promoting consistency in the interpretation and reporting of images. The lesions are classified into six categories, similar to the O-RADS MRI classification. O-RADS USG applies to all non-physiological abnormalities (AL). In contrast, for physiological entities, O-RADS applies to high-risk patients and cases where discrepant findings are reported on another modality, such as CT or MRI [12].

Scores 1, 2, and 3 are given to AL less than 3 cm, 3-10 cm, and more than 10 cm in dimensions, respectively. Further demarcation among score 3-5 lesions was based on locularity, wall thickness, papillary projections, solid component, and color score (CS) [3]. A score of 0 is assigned to incompletely evaluated or non-visualized lesions, except for premenopausal patients with a known genetic predisposition for ovarian malignancy, follow-up of previously visualized lesions, and assessment on an alternative modality [11]. Score 1 denotes physiological findings like follicle or corpus luteal cyst up to 3 cm in diameter. Scores 2 are considered benign in almost all cases, as they carry a risk of malignancy of less than 1%. These lesions range from simple cysts to complex, unilocular to multilocular cysts, up to 10 cm in diameter [11]. Score 3 lesions exhibit unilocular to multilocular morphology, similar to score 2 lesions. They measure less than 10 cm if unilocular or bilocular, and more than 10 cm if multilocular. Solid lesions with shadowing and a CS of 1-3 also fall under this category [11]. These lesions bear a malignant potential of less than 10%. Score 4 lesions are usually bilocular to multilocular in morphology, with an associated non-shadowing solid component, and a CS of 1-3. Score 5 lesions carry the highest malignant potential, exceeding 50%. These lesions are characterized by bilocular or multilocular morphology with an associated solid component showing a CS of 3-4. The presence of ascites or peritoneal nodules is also assigned to this category, regardless of the lesion's morphology. In this 2022 version, a proposal was made to differentiate between hydrosalpinx and hemosalpinx by describing an anechoic, fluid-filled tubular structure [11]. On the lines of the TSIC used for MRI, a scoring system was also devised for CEUSG, with scores of 2, 1, and 0 assigned, respectively, to early, simultaneous, and late enhancement compared to the reference myometrium [1]. Similarly, the quantification of enhancement was also performed, with scores of 3, 2, 1, and 0 assigned to higher, equal, lower, or no enhancement compared to the myometrium.

Color Score

The CS, developed by the International Ovarian Tumor Analysis (IOTA) group, grades vascularity into scores 1-4, denoting no, minimal, moderate, and strong flow, respectively. CS applies only to solid or solid-appearing lesions, as well as multiloculated lesions with and without a solid component [12]. In lesions devoid of a solid component, CS is assessed in the wall and septae.

The original O-RADS USG was revised in 2022, addressing perceived complexity while neglecting the significance of shadowing in solid AL, applicability to patients with normal or obscured ovaries, and discrepancies with O-RADS MRI and SRU guidelines [11,50]. In addition, clarification was sought regarding why the lower malignant potential in solid smooth lesions showed shadowing and bilocular smooth cysts, as observed in the study by the IOTA group. Indeed, solid lesions with marked hypoechogenicity should undergo MRI or CEUSG, thereby increasing specificity towards benign diagnosis [1,50].

O-RADS MRI

An MRI scoring system was devised by Thomassin-Naggara et al. in 2013 to evaluate the AL designated as indeterminate on the USG acronym ADNEX. Further extension of this scoring system was updated as O-RADS MRI in 2020, which was based on the same imaging characteristics of MRI as the ADNEX. T1 and T2, weighted sequences with adjunct DWI and PWI, categorized AL into five subcategories based on the likelihood of malignancy, providing a risk score [8,17,35]. One of the most important highlights of the ADNEX MR scoring system was the characteristic enhancement curve of malignant lesions.

O-RADS MRI is a five-tier system for stratifying indeterminate AL to assess the likelihood of malignancy [51]. O-RADS MRI is reported to enhance reproducibility and consistency while reducing interobserver variability due to differences in expertise, supported by strong evidence [17]. O-RADS MRI also provides referring clinicians and patients a clearer perspective on the probability of malignancy, in turn, leading to better counseling [51].

O-RADS MRI is not applicable in cases of acute conditions like ectopic pregnancy, ovarian torsion, pelvic infection, or extra-adnexal origin of a lesion [5,13,31,32,47]. In one study, AL was falsely assigned a higher O-RADS MRI score of 4 due to associated acute torsion.

The application of either scoring system remains challenging, with the primary issue being the acquisition and interpretation of PWI. PWI analysis involves a complex machine-driven mathematical algorithm for calculating perfusion based on the lesion's dynamic SI characteristics. Even non-contrast MRI (NC-MRI) studies showed good agreement in predicting the risk of malignant potential [17,32]. According to a study by Sahin et al., intermediate-category lesions in NC-MRI were comparable to low and intermediate-risk categories of O-RADS MRI, and suspicious lesions in NC-MRI were comparable to the high-risk category of O-RADS MRI [52]. These findings showed excellent agreement for overall diagnosis rather than intralesional morphology, such as septae.

AL was infrequently found indeterminate on USG and was resected to mitigate the possibility of leaving a low-grade or undetected malignancy unattended. The O-RADS MRI score has been shown to accurately categorize indeterminate AL on USG, subsequently allowing for the potential to avoid unnecessary surgical procedures in more than 88% of cases [7,20,53].

Besides surgical morbidity, removal of ovarian tissue can lead to loss of fertility in premenopausal women and mortality, primarily due to the involvement of the cardiovascular and skeletal systems in the postmenopausal age group [20]. On the contrary, lesions more than 10 cm in dimension are difficult to interpret and must be referred for surgical removal to avoid missing malignant lesions [53]. Prompt surgical referral also leads to better management and effective disease control, translating into higher survival rates, especially when referred to a specialized center [20].

Score 0 is assigned to any lesion that could not be assessed due to large lesion size, imaging artifacts, incomplete study, inability to acquire all required sequences, or cases where the recommended protocol is not followed [13,31,33]. In the 2022 version, obscured imaging features relevant to risk stratification were added under this category [11].

Score 1 lesions, characteristically unilocular and containing simple fluid contents like normal ovarian follicles, cyclical changes, small hemorrhagic cysts, or corpus luteum cysts up to 3 cm in size, are highly likely benign, offering an optimistic outlook for patient outcomes [11,12,16,33]. Their lack of solid components, absence of wall enhancement, and thin intralesional septae make the probability of malignancy nearly zero, providing reassurance. Pelvic lesions of extra-adnexal were also assigned a score of 1 [3,5,6]. The Corpus luteum demonstrates characteristic crenulated inner margins with intense peripheral color flow [16].

Score 2 lesions are similar to O-RADS MRI score 1 in imaging characteristics and nature, characterized by simple fluid contents and the absence of solid components or appreciable wall enhancement [16,17,30,35]. These lesions may be T1 hypointense and T2 markedly hyperintense, indicating a fluid nature. T1 and T2 hyperintense with signal suppression represent fat, while the absence of signal suppression depicts blood contents. A subset of these lesions, namely fibroma and fibrothecoma, show a homogenous hypointense signal on the T2 sequence with no appreciable diffusion restriction (T2-DWI dark) [5,17]. These lesions rarely exhibit solid components, and if they are enhanced, the TSIC type guides further assignment of the score as 3-5 [13,27,40]. These lesions may require follow-up, as suggested by the possibility of being mucinous cystadenoma, in addition to the more common diagnoses of fibrothecoma and leiomyoma [2,3,6]. A case of metastasis from invasive lobular breast carcinoma also showed this double dark morphology [40]. Hydrosalpinx and para-ovarian cysts are placed under this score.

Score 3 lesions are similar to O-RADS MRI score 2 lesions because they can appear unilocular with hemorrhagic contents with a size beyond 10 cm [15]. In addition, these lesions can demonstrate hemorrhagic, mucinous, or proteinaceous contents, recommending a signal assessment of every lesion with fluid-like contents [30,35]. A thin intralesional septae gives a multilocular appearance with a T2 hypointense solid component, which may not enhance. If it is enhanced, it requires further evaluation with the TSIC and should demonstrate TSIC type 1 [35]. In the 2022 version, a new term, biloculated lesion, is introduced for lesions with single intralesional septa and is placed under score 2 [11]. The multilocular lesions are assigned a score of 3. Multilocular cystic morphology is commonly seen in benign lesions [34]. Only one of the 77 lesions scored 3 was malignant in one of the studies [35]. Hydrosalpinx has prominent folds and is a complex fluid of hemorrhagic or pyogenic nature that falls under this score [13]. In the 2022 version, a proposal was made to differentiate between hydrosalpinx and hemosalpinx by describing an anechoic, fluid-filled tubular structure [11].

A solid non-hyperechoic component, avascular nodule, or septae less than 3 mm, and a TSIC type 2 characterize score of 4 lesions [3]. These lesions are considered indeterminate and have variable malignant potential. These lesions may exhibit a high signal on T1 fat-suppressed images, indicating the presence of intralesional blood products [17]. These lesions contain a significantly larger amount of fat than lesions of score 2 [30].

Wong et al., based on their study, suggested categorizing score 4 lesions into high- and low-risk groups. Lesions with papillary projections and CA-125 levels greater than 35 U/mL were assigned to the high-risk subgroup, and those with irregular projections and non-elevated CA-125 levels were assigned to the low-risk subgroup [23].

Score 5 lesions are large, multiseptated lesions with septae or wall thickening greater than 3 mm, exhibiting enhancing solid components of type 3 TSIC and peritoneal carcinomatosis [3,17]. No benign lesions are found for this score.

The O-RADS MRI score exhibits specificity of up to 90% in the overall population, with the highest sensitivity for lesions with scores of 4 and higher [4,12]. O-RADS USG and O-RADS MRI both show high sensitivity for detecting malignant AL at 95%, with O-RADS MRI also showing higher specificity, up to 90%. The lower specificity of O-RADS USG can be partially attributed to its higher sensitivity, which helps prevent the potential for missing malignant lesions. The diagnostic performance of the O-RADS system for benign lesions is relatively low, whereas it shows higher performance in borderline and malignant lesions [6,51,54].

ADNEX

Risk stratification and management have been defined for five different ADNEX risk categories and simultaneously compared with O-RADS [5,44]. O-RADS score of 2 corresponds to a risk of less than 1%, with scores of 3, 4, and 5 indicating risks of 1-10%, 10-50%, and greater than 50%, respectively. O-RADS scores of 0 and 1 lesions are excluded from the ADNEX system [28]. The presence of a solid component, internal septations, more than three papillations, large lesion size (6-8 cm or greater), unilateral presentation, ascites, and a color Doppler vascularity score of 4 indicate a high probability of malignancy. In contrast, acoustic shadowing and a CS of 1 favor a benign etiology, with less than 10% malignant potential [11,28,34,50].

The management of ADNEX risk category 1 AL is not required. Category 2 ALs require further imaging assessment by an expert; category 3 and category 4 ALs may be managed by imaging or referral to a gynecologist or oncologist. Category 5 lesions require an oncology referral.

The most frequent malignant lesions were borderline as well as invasive primary malignancy and metastasis to the ovary. Common benign counterparts were cystic teratomas and mucinous and serous cystadenomas. CA-125 levels do not affect the performance of the ADNEX system; however, they help distinguish between stage 1 disease and stages 2-4 disease.

The Impact of an MR Scoring System on the Therapeutic Strategy for Pelvic Adnexal Masses, also known as the ASCORDIA protocol, was initiated in 2016 and enrolled 377 patients. It is designed to avoid unnecessary surgeries for benign lesions while promptly managing malignant lesions with the goal of curative resection [14,27]. According to this protocol, lesions were divided into three subgroups based on their dimensions: those less than 4 cm, those between 4 and 6 cm, and those greater than 6 cm. In all three subgroups, lesions with an ORADS MRI score of 3 or less were recommended to undergo MRI imaging. A score of 4 required diagnostic resection, and a score of 5 required cytoreductive surgery. Exceptions to this protocol include dermoid cysts and endometriomas measuring 4-6 cm, which are assigned a score of 4; those larger than 6 cm are assigned a score of 2. The size of residual lesions after cytoreductive surgery is one of the most important prognostic factors [19].

Another trial, named MORC, was initiated, which enrolled 645 patients and evaluated AL using CT and MRI [14,27]. CT will be undertaken first, and based on the findings, a treatment plan will be devised and noted. Once treatment is established, the same surgical team will receive an MRI. If the MRI changes the original plan devised after the CT scan, this will also be noted. The treatment plans include follow-up, less aggressive fertility-preserving surgery, cytoreductive surgery, and chemotherapy. Extensive surgery for benign or borderline lesions and failed attempted surgery in patients with extensive disease were deemed inappropriate surgical treatments.

Approach

For O-RADS, USG AL is placed into three categories: physiological, classic benign lesion, and lesion. The physiological category comprises follicle and corpus luteum less than 3 cm in a patient of premenopausal status, assigning a score of 1. The term "cyst" should not be used to avoid ambiguity. A non-physiological entity should be considered if the size exceeds 3 cm [12].

Classic benign lesions include hemorrhagic cysts, endometriomas, and dermoid cysts of ovarian origin, which are further evaluated clinically and by imaging. These lesions show a characteristic appearance on USG and are devoid of internal blood flow. Classic benign lesions of less than 10 cm in size fall under score 2, and those more than 10 cm in size fall under score 3 [12]. Para ovarian and peritoneal inclusion cysts, as well as hydrosalpinx, being extra ovarian entities, once established, complete the assessment and are designated a score of 2 [16].

The lesion category included five subcategories: solid or solid-appearing, unilocular cystic with and without a solid component, and multilocular cystic with and without a solid component. If the outer contour is irregular in solid lesions, the lesion is assigned a score of 5 straightforwardly. In lesions with smooth outer margins, a further score is assigned according to the CS, with no appreciable flow assigned a score of 3, moderate flow assigned a score of 4, and marked flow assigned a score of 5 [12].

For unilocular cystic lesions without solid components, smooth inner wall, and anechoic or echogenic fluid contents, score 2 is assigned for those less than 10 cm and score 3 for those more than 10 cm. Unilocular cystic lesions with an irregular inner wall are assigned a score of 3, irrespective of their size. For multilocular cystic lesions without a solid component, a smooth inner wall warrants evaluation with CS, with strong flow giving the lesion a score of 4 in those larger than 10 cm and 3 in those smaller than 10 cm in size. With irregular inner wall lesions, the lesion is assigned a score of 4, irrespective of size [12].

For unilocular cystic lesions with solid components, a score is assigned depending upon papillary projections; lesions with 1-3 projections are assigned a score of 4, and those with 4 or more projections are assigned a score of 5. For multilocular cystic lesions with a solid component, a CS of 1-2 is designated a score of 4, and a CS of 3-4 gives a score of 3-4 [12].

Fluid confined to the pouch of Douglas is considered physiological; any fluid beyond this is labeled as ascites. Unexplained ascites and peritoneal nodularity upgrade score 3 and 4 lesions to O-RADS score 5 status. Ascites in scores 1 and 2 lesions should prompt investigation for alternate non-malignant entities [12].

One approach for evaluating AL using O-RADS MRI is proposed by Melamud et al., with a similar approach suggested by Suarez-Weiss et al. [13,30]. In this algorithm, the first and foremost observation to look out for is the presence of peritoneal or omental deposits, and once detected, the lesion is assigned a score of 5. Without deposits, if findings conform with a physiological observation, the lesion is assigned a score of 1; otherwise, it is further assessed for scores 2-4.

In the presence of a solid component that appears hypointense on T2 and DWI sequences, the lesion is assigned a score of 2. Otherwise, depending on the type (1, 2, or 3) TSIC curve, the lesion is assigned a score of 3, 4, or 5, respectively. In the absence of TSIC, enhancement of a lesion less than or more than the outer myometrium assigned a lesion a score of 4 and 5, respectively.

If the lesion lacks a solid component, the lesion is designated a score of 2 or 3. Unilocular lesions without peripheral wall enhancement and named lesions of a simple cyst, dermoid, and endometrioma are given a score of 2 [31]. Lesions with peripheral wall enhancement and multilocation are assigned a score of 3 [13].

Another approach was suggested by Thomassin-Naggara et al., which started with identifying the abnormality as a true AL. If the findings remain negative, a score of 1 is assigned. If the AL is confirmed positively, a search for peritoneal disease is conducted, and the lesion is assigned a score of 5.

In the absence of peritoneal or omental deposits, lesions containing appreciable fat components devoid of solid tissue, peripheral wall enhancement, and serous or endometriotic contents are assigned a score of 2. Lesions with mucous or purulent contents and those devoid of solid-enhancing tissue were given a score of 3.

Once solid enhancing tissue was identified, the lesion was assigned a score of 3, 4, or 5, depending on whether it demonstrated a TSIC 1, 2, or 3 pattern, respectively. If TSIC is not feasible, comparative enhancement of the lesion, less than or more than the outer myometrium, gives the lesion a score of 4 and 5, respectively. The only instance where a lesion with a solid component was assigned a score of 2 is when solid tissue is hypointense on both T2 and DWI sequences [32].

A third approach, suggested by Thomassin-Naggara et al., involves identifying the adnexal origin of the lesion as a first step [4]. If confirmed, the lesion is further evaluated for solid components, particularly on T2-weighted, DWI-ADC, and post-contrast sequences, with the latter for assessing TSIC. Purely cystic, fatty, or endometriotic lesions, as well as those without any enhancement and T2-DWI dark lesions, are categorized as score 2. Lesions with proteinaceous or hemorrhagic contents and those with solid components bearing TSCI type 1 are assigned a score of 3. Lesions with solid components demonstrating TSIC types 2 and 3 are assigned scores of 4 and 5, respectively. Peritoneal or omental involvement is assigned a score of 5 regardless of lesional morphology [6].

The fourth approach, suggested by Sadowski et al., involves confirming the adnexal origin of the mass lesion as the initial step, followed by evaluating the presence of any enhancement within the lesion. Furthermore, the lesion is examined for its fat content. A lesion with no enhancement, irrespective of fat content, is assigned a score of 2. Fat-containing lesions with a large enhancing solid component are assigned a score of 4. Lesions with discernible solid components require evaluation with DWI-ADC and TSIC to assign a score of 3, 4, or 5. In lesions devoid of a fat or solid-enhancing component, the mere presence of wall enhancement further scores the lesion, guided by the nature of the fluid. Simple or endometriotic fluid scored 2, whereas hemorrhagic and proteinaceous components scored 3 [27].

Incidentally Detected Lesions

According to the American College of Radiology's revised guidelines issued in 2020, AL measurements of less than 3 cm in premenopausal patients and less than 5 cm in postmenopausal patients are not followed up [16]. Lesions greater than 10 cm in both patient groups warrant prompt characterization by either USG or MRI [3].

The SRU 2019 update recommends follow-up of lesions greater than 5-7 cm in premenopausal patients and greater than 3-5 cm in postmenopausal women [16].

Lesions measuring 5-10 cm in premenopausal patients and 3-10 cm in postmenopausal patients, which have already been evaluated on USG or MRI, are followed up at 6- to 12-month intervals with USG [16]. This size threshold for follow-up lesions is further increased in lesions that are considerably evaluated with DCE-MRI. Similarly, the management of a hemorrhagic cyst is categorized into two stages: early and late, with age ranges of 50-55 years and over 55 years, respectively [11,16]. A 10%-15% change in lesion dimension guides a follow-up strategy with reduction, no change, and an increase in dimensions, suggesting no follow-up, a 12-month follow-up, and a 12-24 month follow-up, respectively [11]. In the premenopausal age group, a hemorrhagic cyst smaller than 5 cm in size requires no further follow-up. Lesions more than 5 cm in premenopausal and of any age in postmenopausal women require follow-up imaging [16].

The guidelines mentioned above do not apply if the lesions are symptomatic, have remained stable over the last two years, or if the patient belongs to a high-risk category based on their history or genetic susceptibility [16].

Malignant Potential

Generally, AL is devoid of solid components or thickened intralesional septae, and a lack of wall enhancement is considered benign. Multiple studies have confirmed that O-RADS MRI scores 1 and 2 lesions show no imaging findings, indicating a malignant nature at presentation or on follow-up, with a malignant nature of less than 0.5% for score 2 lesions [3,13,35]. A study found that scores 1 and 2 lesions had a malignant potential of 0% and less than 1%, respectively. In the same study, the malignant potential of score 3 lesions varied from 3% to 6%, with an average of 5%. Notably, higher values were observed for lesions with solid components. Similarly, scores 4 and 5 lesions were malignant in 50%-90% of cases, with higher values in score 5 lesions [2,3,6,54]. The malignant potential of the O-RADS MRI score of 3 lesions was from 1%-2% to up to 5%, the score of 4 lesions was nearly 50%, and the score of 5 lesions was 90%-100% [5,13,18,35].

O-RADS MRI score 4 lesions have variable false-positive rates for malignancy, ranging from 5% to 90% [23,35,46,55,56,57]. Among these, a study by Aslan and Tosun cited a 50% false-positive rate attributed to a lower temporal resolution resulting from an increased time interval in scanning dynamic sequences, necessitating newer MRI characteristics [35]. O-RADS MRI scores of 5 lesions are malignant in almost all cases, with an average risk of 90%, and require prompt referral to a gynecologic surgeon [1,35]. Pelvic inflammatory diseases (PID), such as those caused by tuberculosis or other bacterial infections, account for the remaining 10% of the risk [32].

Individual lesions

Follicle and Corpus Luteum Cysts

Follicle and corpus luteum cysts are considered physiological findings measuring up to 3 cm in size. A follicle contains clear fluid, whereas a corpus luteal cyst may contain complex fluid of a hemorrhagic nature, thick irregular walls, diffusion restriction, and post-contrast enhancement [13,33].

Hemorrhagic Cyst and Endometrioma

A typical hemorrhagic cyst is a classically unilocular avascular lesion with an internal reticular pattern or the presence of an eccentric retracted blood clot. Both hemorrhagic cysts and endometriomas contain blood products of various stages, typically appearing hyperintense on T1 and T2 sequences during the subacute phase. The endometriotic fluid exhibits additional features, including homogeneous T1 hyperintensity, T2 shading, and dark spot signs [13,31,33]. Peripheral punctate hemorrhagic foci, representing cholesterol deposits, bear high specificity for diagnosing endometrioma by ultrasound, in addition to loculated avascular morphology similar to that of hemorrhagic cysts [11].

Cystadenofibroma (CAF)

CAF is a benign ovarian neoplasm with the most common histological subtype being serous. Typical imaging features are of a multiseptated solid cystic lesion. The serous component, being the most prevalent, also exhibits the highest variability in solid components and septations, as well as T2 low SI and O-RADS MRI scores of 2 and 3 in the majority. The majority of lesions showed a TSIC type 1 curve [36]. The literature described a novel "black sponge" appearance of type 1 CAF showing intense postcontrast enhancement [36].

Germ Cell Tumors

A dermoid cyst is the most common ovarian germ cell tumor seen around the age of 35 [2]. On USG, a dermoid cyst typically shows few locules, with hyperechogenicity of focal, diffuse, or linear morphology, accompanied by posterior shadowing [11]. The lesion shows a heterointense signal on T1 and T2 sequences with suppression of fatty components. They exhibit variable diffusion restriction and postcontrast enhancement depending on the characteristics of the solid components. Mature benign histological type shows larger areas of calcification and fat attenuation than immature and malignant types [17]. A Rokitansky nodule, representing a small solid component, will categorize the lesion as score 2. Conversely, when the solid component is relatively large, the lesion falls under the score of 4 [13,31,39].

Sex Cord Stromal Tumors

Fibroma is the most common benign variant, characterized by a low signal on the T2 sequence, giving a black, sponge-like appearance, a low signal on DWI, and mild postcontrast enhancement on the delayed phase [17]. Sertoli-Leydig and granulosa cell tumors are malignant counterparts that exhibit diffusion restriction, accompanied by post-contrast enhancement of solid components. These tumors also manifest endocrine manifestations in the form of estrogen or androgen excess [17].

Struma Ovarii

Struma ovarii is a rare subtype of ovarian teratoma characterized by a predominant component of thyroid tissue. The typical appearance is that of a multiloculated lesion with a hyperdense area within, the latter representing thyroid tissue. There is also the variable presence of calcification and fat with postcontrast enhancement of the septae and solid components. The solid component of the lesion exhibits intense and early postcontrast enhancement attributable to high iodine content [49].

Epithelial Lesions

These are serous or mucinous cystadenomas or CAFs, comprising the largest subgroup of ovarian tumors. These epithelial variants are predominantly cystic, with or without internal septations, and contain variable solid components. The borderline epithelial variants display prominent solid components with papillary projections, which show TSIC type 2 or 3 curves on postcontrast dynamic imaging [17]. CEUSG findings also revealed a marginal higher enhancement in borderline papillomas compared to their benign counterparts [1]. The invasive epithelial variants appear similar to borderline epithelial lesions, exhibiting the presence of malignant ascites, large lymphadenopathy, and peritoneal implants, as well as significantly elevated levels of tumor markers [17].

Metastasis

Any lesion in the contralateral ovary, ascites, peritoneal or omental deposits, and abdominal lymphadenopathy are all considered metastatic lesions [17,35]. DWI is the best single sequence for detecting peritoneal metastasis [17].

Pitfalls

On the downside, the O-RADS MRI score was misclassified in approximately 9% of cases when applied to USG indeterminate lesions. The maximum errors were made for score 4 lesions, nearly 50%, with the most common misinterpretation in solid tissue analysis [26,30,33,35]. A minority of misinterpretations were also attributed to PID, characterized by intense postcontrast enhancement and diffusion restriction secondary to an inflammatory response and the presence of pus, respectively [33].

In the context of the O-RADS MRI score, a major misinterpretation involved designating lesions with a score of 3 or less as borderline or malignant and classifying lesions with a score of 4 or 5 as benign [40].

The major categories of misinterpretation can be segregated into failure to identify and characterize. Failures of identification omitted the lesion entirely and misinterpreted an extra adnexal lesion as adnexal, especially when the size was more than 5 cm [33]. Extra adnexal lesions encountered were fibroids, peritoneal hematoma or cyst, neurogenic tumors, and adjoining primary colorectal and urinary bladder malignancies. A case of benign peritoneal thickening was also interpreted, and peritoneal metastasis was assigned a score of 5. Gonadal vessels leading to AL highly suggest the adnexal origin of the lesion [30,33].

Obturator lymph nodes may also be mistaken for normal postmenopausal ovaries, with the internal iliac vessels serving as landmarks to differentiate between them.

Failure to characterize was due to a multitude of reasons, including poor image quality, motion artifacts, inadequate assessment of solid tissue, non-contrast studies resulting from a lack of or atypical TSIC, and non-interpretable DWI-ADC [26]. Poor assessment of solid tissue was contributed to by its small size, T2 hypointense signal, lack of appreciation or misinterpretation of the shoulder and plateau on TSIC, and presence of collision lesions or ongoing torsion. In addition, solid components such as septations, blood clots, fat, debris, endosalpingeal folds, and Rokitansky nodules interfered with assessment, as they are not true solid tissue [33]. Cases of ovarian fibroma, cystadenofbroma, and endosalpingeal folds displayed TSIC type 3, falsely elevating the O-RADS score to 4 and 5 [6].

If individual lesions are accounted for, DCE-MRI misclassifies mature teratoma as scoring 4 or 5 [7]. In a non-DCE MRI study, mature teratomas were correctly assigned a score of 2. Additionally, the presence of a large solid tissue in fatty and benign lesions, such as Brenner tumor and adenofibroma, falsely elevated the score to 4 or 5 [7].

Limitations

The risk categorization in O-RADS depends on the modality used, namely USG or MRI. USG, a widely available and cost-effective modality without the risk associated with gadolinium administration, takes the upper hand in guiding the O-RADS score in a larger proportion of the population [57]. This difference in designated O-RADS scores is predominantly due to the O-RADS MRI scoring system's nonspecificity in lesions containing fat, with scores of 2 and 4 assigned to lesions containing small and large intralesional fat, respectively [30,47]. O-RADS MRI also lacks recommendations based on the lesion score [30].

Additionally, mucinous tumors can be assigned a lower O-RADS score if they have a small solid component. The contribution of advanced imaging techniques, including contrast enhancement for TSIC assessment and diffusion-weighted imaging in fat-containing lesions, has also not been established.

Due to variations in magnetic field homogeneity, the results of DWI-ADC and TSIC can differ between magnetic strength machines, specifically 1.5 Tesla and 3 Tesla scanners [8]. DWI-ADC interpretation can be limiting in normal ovarian mesenchyme, highly cellular benign AL, and highly differentiated hypovascular malignant AL [34].

A further objective evaluation by DWI-ADC to determine specific values can help stratify AL into surveillance and surgical groups, potentially impacting outcomes [21].

On this basis, Hottat et al. evaluated the comparison of ADC values between entire lesions, as delineated by three-dimensional delineation, and only solid components, as delineated by two-dimensional delineation, considering the commonly encountered heterogeneity of AL [42,43].

TSIC has the limitation of technical expertise to perform it accurately and is non-feasible in patients who have undergone a hysterectomy in the past for any reason, although a type 1 curve can still be well assessed [27]. In such cases, the demarcation between TSIC 2 and 3 is not feasible, which prevents a confident distinction between O-RADS MRI scores 4 and 5 lesions [8,27,33]. In cases of hysterectomy, TSIC should be evaluated for a shoulder, with an absence assigned as a low-risk curve. In contrast, the presence of a shoulder designates the curve as intermediate or high risk [30]. The TSIC type 2 curve significantly overlaps benign, malignant, and inflammatory lesions, warranting further investigation and corroboration with DWI-ADC, MRS, and other techniques. Also, difficulty correctly interpreting shoulder and plateau segments can lead to categorizing a TSIC as 1 or 2 [8,33].

The optimal cutoff value of mean ADC values in correlation with the O-RADS MRI score is not widely established, especially for 3 Tesla and higher magnetic field strength systems [41]. The contribution of tumor marker levels, such as CA-125, family history of ovarian malignancy, personal history of malignancy in another organ system, and menopausal status, has not been investigated in many studies [4]. CA-125 levels of more than 35 U/ml were considered elevated, indicating malignant etiology [6].

The validity of MRS depends on the demonstration of clear metabolite peaks and the exclusion of irrelevant or spurious peaks [34]. The value of MRS is established in conjunction with the morphological features of AL and DWI-ADC, although a direct correlation between MRS and TSIC has not been established due to a lack of data [34]. A choline peak has also been found in benign AL, which exhibits a high proliferative capability. No specific cutoff value for the choline peak has been established to differentiate between benign and malignant lesions [34].

In patients with two or more ALs, bias can be generated when both lesions are assessed together rather than individually. Finally, the NPV of O-RADS MRI cannot be ascertained in cases without an AL [6]. The higher occurrence of positive cases detected in various studies can be attributable to most establishments being tertiary care centers and most radiologists bearing a high level of experience [26].

APTW-MRI is a promising technique for differentiating benign from malignant lesions based on the protein concentration of the cystic component. Further studies are required to assess its applicability in patients who cannot be administered gadolinium contrast for any reason [44]. Finally, language model chatbots showed some potential for reporting and categorizing AL based on the O-RADS MRI model [58].

## Conclusions

The O-RADS scoring system is a valuable tool for radiologists in assessing ALs, aiding both less experienced and expert professionals by enhancing diagnostic confidence, especially for USG, where its sensitivity is higher than its specificity. Proper use of O-RADS, in conjunction with accurate preoperative imaging, can prevent unnecessary surgical explorations and has advantages in identifying benign ALs. While O-RADS is not applicable for acute symptoms, its key role is to differentiate between score 2-3 ALs, which can be closely followed or undergo minimally invasive surgery, and score 4-5 lesions, requiring prompt referral to a gynecologist or oncologist.

Though MRI O-RADS can be subjective, objective assessments are possible with DWI-ADC, TSIC, and MRS, with TSIC being preferred for its objectivity in grading scores 3-5. Classic USG appearances often obviate the need for an MRI, which is more useful for problem-solving or characterizing cases with high suspicion of metastasis. Identifying lesions by name, in addition to an O-RADS score, is recommended. Overall, O-RADS is an evolving stratification system that radiologists should use in conjunction with other imaging findings, clinical presentation, patient history, and laboratory parameters to inform their conclusions. Its widespread adoption is expected to improve diagnostic accuracy, reproducibility, and communication, leading to more prompt management. Future studies are needed to refine the system, particularly to reduce false positives and enhance the recognition of specific lesion types.
